# Effects of intra-operative administration of subanesthetic s-ketamine on emergence from sevoflurane anesthesia: a randomized double-blind placebo-controlled study

**DOI:** 10.1186/s12871-023-02170-5

**Published:** 2023-06-23

**Authors:** Tiantian Liu, Xinxin Zhang, Ao Li, Tingting Liu, Xue Yang, Huanhuan Zhang, Yanling Lei, Qianzi Yang, Hailong Dong

**Affiliations:** 1 Department of Anesthesiology and Perioperative Medicine, Xijing Hospital, Fourth Military Medical University, 127 Changle West Road, Xi’an, 710032 China; 2 Department of Anesthesiology, Ruijin Hospital, Shanghai Jiaotong University School of Medicine, 197 Ruijin Er Road, Shanghai, 200025 China

**Keywords:** Recovery time, s-ketamine, Sevoflurane, EEG, Neurological symptoms

## Abstract

**Background:**

Ketamine is administered in the perioperative period for its benefits in analgesia, anti-agitation and anti-depression when administered at a small dose. However, it is not clear whether the intra-operative administration of ketamine would affect emergence under sevoflurane anesthesia. To investigate this effect, we designed this trial.

**Methods:**

In this randomized, double-blind, placebo-controlled study, we enrolled 44 female patients aged 18–60 who were scheduled to elective laparoscopic gynecological surgeries. All patients were randomly assigned to saline or s-ketamine group. In s-ketamine group, patients received 0.125 mg/kg s-ketamine 30 min after the start of surgery. In saline group, patients were administered the same volume of saline. Sevoflurane and remifentanil were used to maintain general anesthesia. The primary outcome was emergence time. We also assessed postoperative agitation, cognitive function, and delirium. In addition, we collected and analyzed prefrontal electroencephalogram (EEG) during and after general anesthesia.

**Results:**

There were no significant differences in emergence time between s-ketamine group and saline group (10.80 ± 3.77 min vs. 10.00 ± 2.78 min, *P* = 0.457). Neither postoperative agitation (4 [3, 4] vs. 4 [3, 4], *P* = 0.835) nor cognitive function (25.84 ± 2.69 vs. 25.55 ± 2.19, *P* = 0.412) differed between groups. No postoperative delirium was observed in either group. Subanesthetic s-ketamine resulted in active EEG with decreased power of slow (-0.35 ± 1.13 dB vs. -1.63 ± 1.03 dB, *P* = 0.003), delta (-0.22 ± 1.11 dB vs. -1.32 ± 1.09 dB, *P* = 0.011) and alpha (-0.31 ± 0.71 dB vs. -1.71 ± 1.34 dB, *P* = 0.0003) waves and increased power of beta-gamma bands (-0.30 ± 0.89 dB vs. 4.20 ± 2.08 dB, *P* < 0.0001) during sevoflurane anesthesia, as well as an increased alpha peak frequency (-0.16 ± 0.48 Hz vs. 0.31 ± 0.73 Hz, *P* = 0.026). EEG patterns did not differ during the recovery period after emergence between groups.

**Conclusion:**

Ketamine administered during sevoflurane anesthesia had no apparent influence on emergence time in young and middle-aged female patients undergoing laparoscopic surgery. Subanesthetic s-ketamine induced an active prefrontal EEG pattern during sevoflurane anesthesia but did not raise neurological side effects after surgery.

**Trial registration:**

Chinese Clinical Trial Registry, ChiCTR2100046479 (date: 16/05/2021).

**Supplementary Information:**

The online version contains supplementary material available at 10.1186/s12871-023-02170-5.

## Background

Ketamine, an N-methyl-D-aspartate (NMDA) receptor antagonist, was initiated to be used clinically for anesthetic induction and maintenance in the 1970s [1]. However, the high incidence of psychotomimetic side effects of ketamine anesthesia (1–2 mg/kg i.v.), such as dissociation, postoperative cognitive dysfunction, hallucinations and nightmares [2], has seriously limited the application of ketamine. In recent years, subanesthetic ketamine (< 1 mg/kg) [3, 4] has gained clinical attention because of the benefits for postoperative analgesia [5–8], anti-agitation [9–11] and anti-depression [12–14], and no significant increase in psychotomimetic effects has been observed [4, 15, 16]. Recent guidelines suggest the use of a subanesthetic ketamine bolus (up to 0.35 mg/kg) or infusions (up to 1 mg•kg^−1^•h^−1^) as an adjunct to opioids for perioperative analgesia [17]. Furthermore, according to Gorlin AW et al*.*, the ideal dose of subanesthetic ketamine to avoid postoperative side effects, such as dissociative states, is 0.1–0.3 mg/kg as a bolus and 0.1 to 0.3 mg•kg^−1^•h^−1^ as an infusion [18].

Theoretically, the co-administration of subanesthetic ketamine during general anesthesia could deepen the anesthetic depth. A series of studies have confirmed that low-dose ketamine reduces the consumption of propofol [19–21] and the minimum alveolar concentration (MAC) of sevoflurane [22, 23] in surgical patients. However, subanesthetic ketamine also induces a paradoxical increase in bispectral index (BIS) during both propofol and inhalation general anesthesia [24–26]. Nevertheless, the effect of low-dose ketamine on the emergence of patients under general anesthesia is still under debate. Some clinical studies found that perioperative administration of subanesthetic ketamine (< 1 mg/kg) did not change the recovery process from propofol anesthesia, but some confirmed the emergence delay [19, 27, 28]. In fact, Hambrecht-Wiedbusch VS et al*.* reported that subanesthetic ketamine (25 mg/kg intraperitoneally, one-sixth of anesthetic dose) accelerated the emergence of rats after isoflurane anesthesia, although it deepened the anesthetic depth detected by cortical electroencephalogram (EEG) signatures [29]. This dual effects of ketamine on inhalation anesthesia may bring specific benefits to surgical anesthesia management if clinical investigations would provide solid evidence.

S-ketamine (induction dose 0.5–1 mg/kg i.v.), the pure dextrorotatory enantiomer of ketamine, has an approximately two-fold higher sedative potency compared with ketamine, associated with a stronger effect at the NMDA receptor [1, 2]. Because of its higher anesthetic potency and fewer side effects [1, 4, 30], s-ketamine is currently more popular and more easily available in anesthesia clinics than ketamine, at least in Chinese hospitals. In our pilot trial, we found that the administration of subanesthetic s-ketamine (0.125 mg/kg, comparable to 0.25 mg/kg ketamine and one-sixth of the anesthetic dose) could reduce the emergence time of sevoflurane anesthesia by 10.6%. Therefore, we designed this clinical trial to mainly investigate whether the subanesthetic s-ketamine could reduce emergence time of surgical patients from sevoflurane-maintained general anesthesia. To avoid the gender dimorphism in anesthesia emergence [31–34], female patients undergoing laparoscopic gynecological surgeries were only recruited.

## Methods

This prospective, randomized, controlled, double-blind trial was registered in the Chinese Clinical Trial Registry (chictr.org.cn) (ChiCTR2100046479, date: 16/05/2021). Ethical approval for this study (NO. KY20202065-X-1, date: 07/04/2021) was provided by the Medical Ethics Committee of the First Affiliated Hospital of the Fourth Military Medical University. This trial was conducted from May 2021 to September 2021 in the Department of Anesthesiology and Perioperative Medicine of the First Affiliated Hospital of the Fourth Military Medical University. Written informed consent was obtained from all patients before enrollment. The principles of Declaration of Helsinki were followed for this study. This manuscript adheres to the applicable CONSORT guidelines.

### Study population

Han Chinese patients aged 18–60 years old with an American Society of Anesthesiologists (ASA) physical status of I–II who were scheduled for elective laparoscopic gynecological surgery and signed the informed consent voluntarily were eligible for inclusion in this study. Patients with a history of psychiatric disorders or neurological diseases, alcohol or drug abuse, operation within one month before surgery, use of sedative medicine or antidepressant within one week before surgery, abnormal cognitive function, inability to communicate fluently, body mass index (BMI) ≥ 28 or ≤ 18, contraindications to s-ketamine, and who participated in other trials or who once were recruited were excluded from this study. The withdrawal criteria included: operation time of < 1 h or > 3 h, unexpected intraoperative conversion to laparotomy, incomplete cases data, patients admitting to Intensive Care Unit (ICU) after surgery.

### Randomization and blinding

The simple randomization was chosen in our trial which was performed using an online randomization list to ensure that each group contained 22 subjects. The subjects were randomly assigned 1:1 to either the saline group or the s-ketamine group. The randomization envelopes contained grouping information. The nurses who preserved and opened the envelops and prepared the s-ketamine or 0.9% saline were not involved in patient care. S-ketamine was diluted in saline to a total volume of 10 ml. The same volume of saline was used in the control group. Syringes of saline and ketamine were identical. The other investigators and all subjects were blinded to randomization.

### Anesthesia and monitoring

Dexmedetomidine, midazolam, and anticholinergic drugs were not permitted before surgery. Vital signs such as pulse oximetry, blood pressure (BP), and electrocardiography were monitored as soon as the patients arrived in the operating room. A Chinese brand of anesthesia depth monitor based on EEG, ConView system (ConView YY-105, Pearlcare Medical Technology Company Limited, Zhejiang, China), was used. Anesthesia index (Ai) is a digital indicator for anesthesia depth derived from the ConView [35]. However, ConView system was used to collect prefrontal EEG data rather than to guide the anesthesia management. Therefore, the anesthetic depth monitor was covered by an opaque box and the alarms were turned off. The anesthesia was managed by a senior anesthesiologist based on clinical needs and personal experience. Anesthesia was induced with 1–2 mg/kg propofol, 0.2–0.4 μg/kg sufentanil, and 0.6 mg/kg rocuronium, and maintained by continuous infusion of 0.1–0.2 μg•kg^−1^•min^−1^ remifentanil and inhaled sevoflurane (0.8–1.5 MAC). A manual ventilation model with 100% oxygen was used to assist respiration until the tracheal tube was inserted with adequate sedation and muscle relaxation. Before the incision, non-steroidal anti-inflammatory drugs were administered, such as flurbiprofen axetil or parecoxib sodium injection. Ventilation was controlled mechanically to maintain an end-tidal carbon dioxide concentration of 30–40 mmHg and an airway pressure below 30 cmH_2_O. Oxygen and air were mixed in a ratio of 1:1, and the flow rate was 2 l/min. S-ketamine (0.125 mg/kg) or the same volume of saline was administered (i.v.) 30 min after the start of surgery. Muscle relaxants were no longer administered 30 min before the end of surgery, and all patients received a bolus of 2 mg tropisetron. End-tidal sevoflurane was gradually adjusted to 0.8 MAC before the end of surgery and sevoflurane was stopped as soon as the skin was sutured. Meanwhile, ventilation was set at 100% oxygen, 6 l/min flow rate, 14 bpm respiratory rate, and 8 ml/kg tidal volume. During the recovery period, we followed the process of awake extubation. When patients established regular breathing and an adequate spontaneous minute ventilation and can open eyes and obey commands, we extubated the tracheal.

### Outcome measures

The primary outcome was emergence time, which was defined as the interval between the cessation of sevoflurane and the point at which the patient opened their eyes. During this recovery period, we aroused the subjects by calling their names every 30 s until they responded by opening their eyes.

Secondary outcomes included the characteristics of prefrontal EEG spectra across the administration of s-ketamine, Ai (generated by the ConView YY-105), gamma power, gamma peak frequency and agitation score during the recovery period, and cognitive function and delirium assessments one day after the surgery. The agitation level was assessed using the Sedation-Agitation Score (SAS). Delirium and cognitive function were evaluated using the Confusion Assessment Method for the ICU (CAM-ICU) and Mini-Mental State Examination (MMSE), respectively. These scales of assessment used in our trial are all in Chinese, which have been widely used for Chinese in both clinic practice and clinical trials [36–38]. The recovery period was defined as the duration from the cessation of sevoflurane administration to 5 min after extubation.

### Spectral processing

EEG data were obtained at a sampling rate of 500 Hz by using ConView. The ConView system is designed by Pearlcare Medical Technology Company Limited (Zhejiang, China), based on three different parameters of EEG: sample entropy (SampEn), 95% spectral edge frequency (SEF) and burst suppression ratio (BSR). The new anesthetic index named Ai is calculated with the algorithm based on decision tree and least square. Ai ranges from awake (80–99), to light sedation (60–80), general anesthesia during surgery (40–60), deep hypnotic state (< 40) and an isoelectric EEG (0). A multicenter clinical study has confirmed that the performance of Ai as a depth of anesthesia monitor was similar to that of BIS [35].

We analyzed the EEG and Ai value across the period of the administration of s-ketamine and the 2-min EEG epochs that were selected 10 min before and 2–8 min after the administration of s-ketamine or saline in detail. We also analyzed the EEG during recovery period.

The power spectra quantifying the energy in the EEG at each frequency were calculated using the multi-window spectrum algorithm in MATLAB R2018b (MathWorks, Natick, MA). The parameters used for statistical inference were as follows: time-bandwidth product = 3, number of tapers = 5, window size = 2 s, and window overlap rate = 99.5%. Group-level spectrograms were obtained by computing the median power across all subjects at each time point and at each frequency. We also computed the power and peak frequency at the time and frequency bands of interest, respectively. The peak frequency is the frequency with the highest power within a certain frequency band. We obtained the Ai value from ConView YY-105 directly.

The mean power and peak frequency of the EEG signal were calculated for the following frequency bands: slow wave (0.3–1 Hz), delta (1–4 Hz), theta (4–8 Hz), alpha (8–12 Hz), beta (12–25 Hz), gamma (25–50 Hz), and beta-gamma (12–50 Hz).

### Statistical analysis

The sample size was calculated based on the time of emergence. According to the results of our previous study, the emergence time was 7.7 min with a standard deviation (SD) of 1.2. In our pilot trial, we found that the administration of subanesthetic s-ketamine could reduce the emergence time by 10.6%. Then, we hypothesized that the s-ketamine could decrease the emergence time by 15%. Considering 80% power, 20% dropout, and adopting a ratio of 1:1, 22 participants were required in each group.

Statistical analyses were performed using SPSS Statistics 23 (IBM, Armonk, NY) and GraphPad Prism 8.0.1 (GraphPad Software, San Diego, CA). Per-Protocol (PP) analysis was used to analyze all outcomes. According to withdrawal criteria, 5 patients were excluded at the data analysis even though they have received the interventions. The remaining patients will be categorized and analyzed according to their allocation and they have strictly followed the trial procedure. The results are expressed as mean ± SD, median, or n (%). The primary endpoint was compared between the two groups using a two-tailed t-test. For the secondary outcomes, ordinal and continuous data were computed using two-tailed t test or two-way ANOVA corrected with the Bonferroni test, while non-normally distributed data and rates were analyzed with the Mann–Whitney U test and the chi-squared test, respectively. The relationship between Ai values and EEG features was explored by using Pearson’s Correlation. If *P* < 0.05, values were considered statistically different.

## Results

**Fig. 1:**
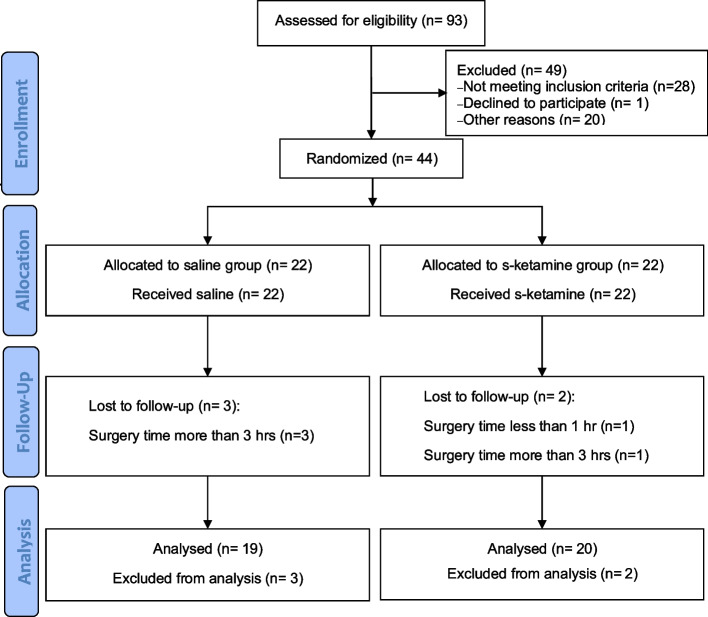
Study flowchart

Forty-four subjects were randomized in our study from May 2021 to September 2021 in the Department of Anesthesiology and Perioperative Medicine of the First Affiliated Hospital of the Fourth Military Medical University. Five of patients were not followed up and excluded according to the withdrawal criteria (the duration of operations lasted > 3 h in four patients and < 1 h in one patient). Thus, 19 subjects in the control group and 20 subjects in the s-ketamine group completed the study and were analyzed (Fig. 1).

The demographics of the patients in the two groups were comparable. No significant differences in ASA classification, baseline cognitive function, duration of surgery or anesthesia, dose of remifentanil or sevoflurane, or incidence of intra-operative hypothermia were found between the two groups (Tables 1 and 2). The anesthesiologists managed the depth of anesthesia based on hemodynamics and their clinical experience. As shown in the Table 2 and Supplementary figure 1, no significant fluctuations in the average values of BP (saline: -2.22 ± 6.39 mmHg vs. s-ketamine: -2.73 ± 6.50 mmHg, *P* = 0.794) or heart rate (saline: -0.10 ± 4.39 bpm vs. s-ketamine: 1.56 ± 5.01 bpm, *P* = 0.250) were observed during the period from 15 min before to 20 min after the administration of s-ketamine.

**Table 1:** Baseline characteristics

	Saline Group (*n* = 19)	S-ketamine Group (*n* = 20)	*P*
Age (yr)	44.37 ± 9.74	46.25 ± 6.77	0.486
Height (cm)	159.40 ± 7.03	160.50 ± 4.33	0.570
Weight (kg)	58.16 ± 6.00	57.45 ± 7.78	0.753
BMI (kg/m^2^)	22.92 ± 2.60	22.68 ± 2.81	0.778
ASA class			0.605
I	1 (5.26)	3 (15.00)	
II	18 (94.74)	17 (85.00)	
Baseline MMSE	26.68 ± 1.86	26.35 ± 1.81	0.573

Data are presented as mean ± SD or n (%)

*Abbreviations: BMI* Body mass index, *ASA* American Society of Anesthesiologists, *MMSE* Mini-Mental State Examination

**Table 2:** Primary outcome and intraoperative data

	Saline Group (*n* = 19)	S-ketamine Group (*n* = 20)	*P*
Emergence time (min)	10.80 ± 3.77	10.00 ± 2.78	0.457
Duration of surgery (min)	117.00 ± 29.80	99.52 ± 31.27	0.083
Duration of anesthesia (min)	156.90 ± 34.23	140.10 ± 35.23	0.141
Total remifentanil (μg)	1281.00 ± 317.70	1177.00 ± 400.80	0.375
Total sevoflurane (ml)	28.40 ± 8.40	25.64 ± 7.96	0.299
Δ blood pressure (mmHg)	-2.22 ± 6.39	-2.73 ± 6.50	0.794
Δ heart rate (bpm)	-0.10 ± 4.39	1.56 ± 5.01	0.250
Hypothermia	6 (31.58)	5 (25.00)	0.652

Data are presented as mean ± SD or n (%). Δ blood pressure and Δ heart rate were obtained by subtracting the pre-administration values from the post-administration values. Hypothermia: core temperature is below 36 degrees

### Primary outcome

The time from the cessation of sevoflurane administration to the emergence of patients in the s-ketamine group was not significantly different from that in the saline group (saline: 10.80 ± 3.77 min vs. s-ketamine: 10.00 ± 2.78 min, *P* = 0.457) (Table 2).

### Secondary outcomes

#### Neurological symptoms

There were no notable differences in SAS scores (saline: 4 [3, 4] vs. s-ketamine: 4 [3, 4], *P* = 0.835) between the two groups during the recovery period. One day after the operation, MMSE scores in the s-ketamine group resembled those in the saline group (saline: 25.84 ± 2.69 vs. s-ketamine: 25.55 ± 2.19, *P* = 0.412), and none of the patients in either group developed delirium (Table 3).

**Table 3:** Secondary outcomes

	Saline Group (*n* = 19)	S-ketamine Group (*n* = 20)	*P*
SAS	4 (3, 4)	4 (3, 4)	0.835
Postoperative MMSE	25.84 ± 2.69	25.55 ± 2.19	0.412
Delirium	0	0	

Data are presented as mean ± SD, median (interquartile range) or n (%)

*Abbreviations: SAS* Sedation-agitation Scores, *MMSE* Mini-Mental State Examination

#### Prefrontal EEG spectra

**Fig. 2:**
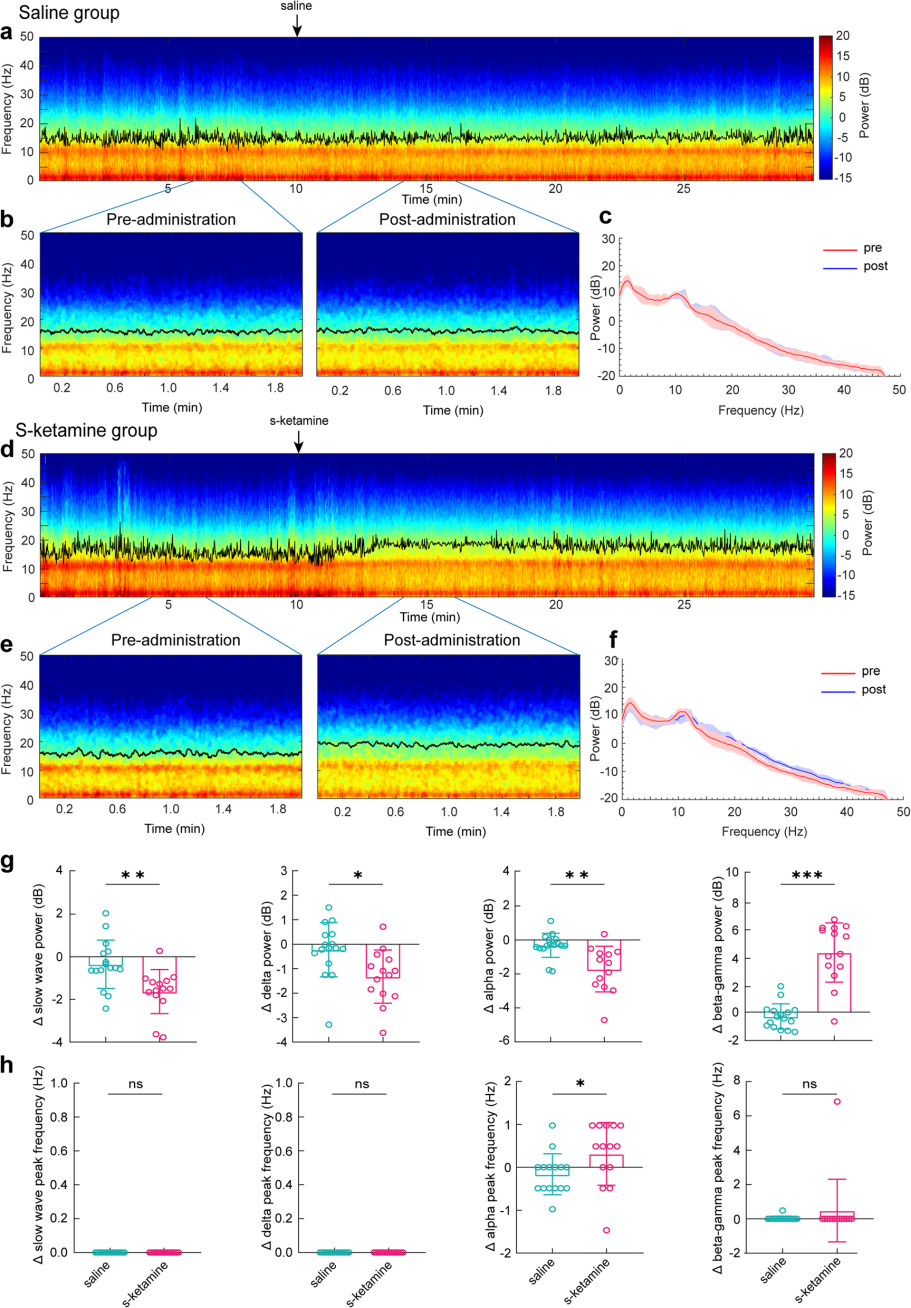
Spectral analysis of EEG signals before and after the administration of s-ketamine. **a**, **d** Median spectrograms of EEG pre- and post-administration of saline or s-ketamine during sevoflurane anesthesia. **b**, **e** 2-min epochs for analyzing. Black lines represent the edge frequency. **c**, **f** The power spectra of 2-min EEG pre- and post-administration of saline and s-ketamine, respectively. **g** The changes of power at slow wave, delta wave, alpha wave and beta-gamma wave (from left to right) between saline and s-ketamine groups. The power of slow wave, delta and alpha bands decreased but beta-gamma waves increased after the administration of s-ketamine. **h** The changes of peak frequency at slow wave, delta wave, alpha wave and beta-gamma wave (from left to right) between saline and s-ketamine groups. The changes of peak frequency of slow wave, delta and beta-gamma in saline group were not different from that of s-ketamine group, but alpha wave increased significantly after the administration of s-ketamine. The changes of power and peak frequency were obtained by subtracting the pre-administration values from the post-administration values. Beta-gamma: the frequency band of beta and gamma. ^*^P < 0.05, ^**^P < 0.01, ^***^P < 0.001 saline group vs. s-ketamine groups

To evaluate the intra-operative effect of subanesthetic s-ketamine, we exhibited the full-range EEG median spectrograms of saline and s-ketamine groups (Fig. 2a, d), in which the edge frequency was significantly increased within 5 min after the s-ketamine administration. These active changes in EEG lasted for about 10 min. An increase of power in the high frequency band after s-ketamine administration was clearly seen in the 2-min spectrograms (Fig. 2b, e). Furthermore, the power of the frequency band between 15 and 50 Hz was significantly increased after s-ketamine administration (Fig. 2f), while saline did not induce significant differences in the power spectrum (Fig. 2c). Specifically, the spectral power in the slow (saline: -0.35 ± 1.13 dB vs. s-ketamine: -1.63 ± 1.03 dB, *P* = 0.003), delta (saline: -0.22 ± 1.11 dB vs. s-ketamine: -1.32 ± 1.09 dB, *P* = 0.011), and alpha bands (saline: -0.31 ± 0.71 dB vs. s-ketamine: -1.71 ± 1.34 dB, *P* = 0.0003) (Fig. 2g) was markedly decreased in the s-ketamine group compared with that in the saline group, whereas the power of the beta-gamma band (saline: -0.30 ± 0.89 dB vs. s-ketamine: 4.20 ± 2.08 dB, *P* < 0.0001) was obviously elevated after s-ketamine administration (Fig. 2g). The peak frequency in the alpha band (saline: -0.16 ± 0.48 Hz vs. s-ketamine: 0.31 ± 0.73 Hz, *P* = 0.026), but not in the other bands, increased in the s-ketamine group compared with that in the saline group (Fig. 2h). S-ketamine also caused an increase of Ai value (saline: 48.49 ± 7.94 vs. s-ketamine: 57.95 ± 8.45, *P* < 0.001) 5 min after the administration. The increase of Ai then lasted for about 10 min and became comparable to saline controls afterwards (Supplementary Fig. 2a). And the changes of Ai were well related with the changes of EEG features across the administration of subanesthetic ketamine. Negative correlations were observed between the changes of Ai values and the changes of slow wave (*r* = -0.52, *P* = 0.003), delta (*r* = -0.55, *P* = 0.002) and alpha power (*r* = -0.58, *P* = 0.001), and a positive relation was observed between the changes of Ai values with the changes of beta-gamma power (*r* = 0.63, *P* = 0.0002) (Supplementary Fig. 2b). No correlation between the changes of Ai values and the changes of alpha peak frequency was found (*r* = 0.25, *P* = 0.183) (Supplementary Fig. 2c).

**Fig. 3:**
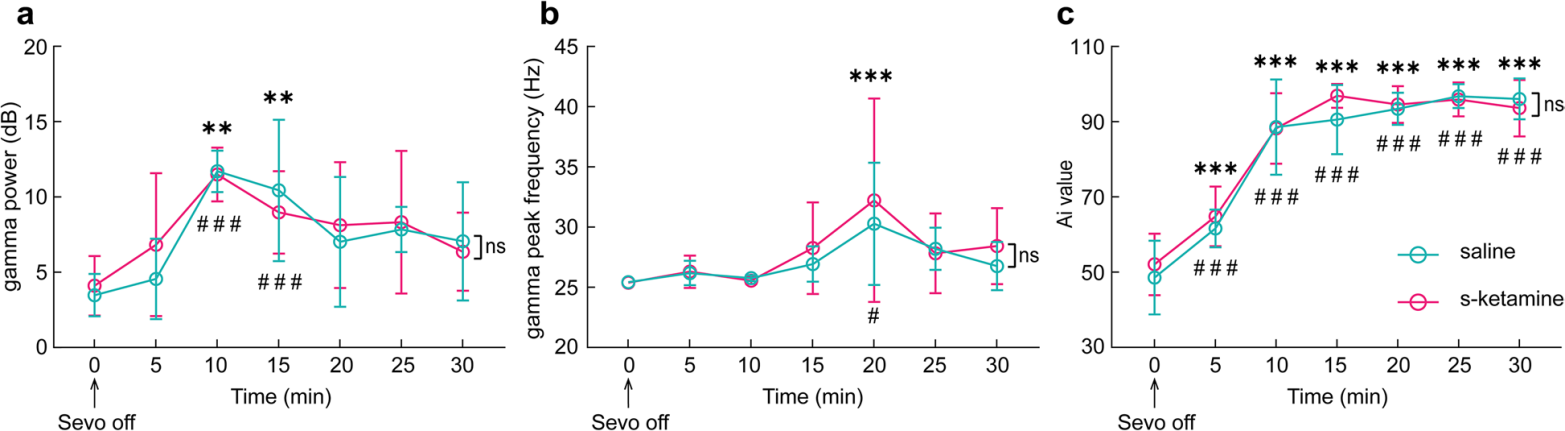
The characteristics of prefrontal EEG signals across anesthesia recovery states. The dynamic changes of gamma power (**a**), gamma peak frequency (**b**) and Ai values (**c**) in the saline group did not differ from that of s-ketamine group but there were evident changes after sevoflurane off compared with before in both groups. ^#^*P* < 0.05 and ^###^*P* < 0.001 in the saline group or ^**^P < 0.01 and ^***^P < 0.001 in the s-ketamine group vs. the time point at sevoflurane cessation. Abbreviations: Sevo = sevoflurane

During the recovery period after the cessation of sevoflurane, the power and peak frequency showed no differences between two groups. The power of gamma oscillations gradually increased and then declined after recovery of consciousness (Fig. 3a). The peak frequency of the gamma band also gradually increased during the recovery but arrived at its peak slightly later than the power of the gamma frequency (Fig. 3b). The Ai value increased reasonably in both groups, and no differences were found between them (Fig. 3c).

## Discussion

In this randomized controlled study, we observed the effects of a bolus of subanesthetic s-ketamine administered during elective laparoscopic gynecological surgery on emergence time in the female patients. We did not find a difference in the time duration from cessation of anesthetics to eye opening between the s-ketamine and saline groups. The agitation level, cognitive function and incidences of delirium, in the s-ketamine group were similar to those in the saline group. However, the administration of subanesthetic s-ketamine induced active EEG spectra with decreased power of slow wave, delta, and alpha bandwidths, and increased power of beta-gamma waves during sevoflurane anesthesia. The alpha peak frequency significantly increased.

A synergistic interaction tends to occur when two or more drugs produce similar effects by different mechanisms [39, 40], and it was naturally considered that perioperative administration of ketamine could deepen the anesthetic depth and increase the emergence time of propofol or sevoflurane general anesthesia. In a study conducted in adult patients under desflurane anesthesia, ketamine administered after induction at a dose of 1 mg/kg delayed the response of patients to verbal stimuli [41]. Similarly, Zhang C et al. reported that the subanesthetic s-ketamine (0.2 mg/kg i.v., comparable to 0.4 mg/kg i.v. ketamine) administered during induction delayed recovery after propofol anesthesia in laparoscopic cholecystectomy [42]. In the current study, we found that a relatively small-dose of s-ketamine (0.125 mg/kg, comparable to 0.25 mg/kg ketamine) had no significant effect on emergence time from sevoflurane anesthesia, which was in line with Moro ET’s report that intra-operative administration of subanesthetic ketamine (0.2 mg/kg) did not change the recovery process from laparoscopic cholecystectomy under propofol anesthesia [27]. Of note, the discrepancy in emergence time from general anesthesia in combination with subanesthetic ketamine or s-ketamine seems closely related to dosage. However, a meta-analysis revealed that a combination of ketamine (0.5–1 mg/kg i.v.) and propofol could reduce the recovery time in comparison with propofol alone in children [43]. Whether the anesthesia is maintained by a single anesthetic agent or a combined method could probably affect emergence as well. For example, although subanesthetic ketamine (0.5–1 mg/kg i.v.) could reduce the recovery time of propofol anesthesia in children, ketamine (0.2–1 mg/kg i.v.) did not affect the recovery time of patients in propofol- dexmedetomidine or propofol- fentanyl anesthesia [43]. And in an animal experiment, Hambrecht-Wiedbusch VS et al. found that administration of subanesthetic ketamine (25 mg/kg intraperitoneally, one-sixth of the anesthetic dose) in rats during isoflurane anesthesia reduced the emergence time by 44% [29]. Emergence acceleration was associated with an increase in cortical acetylcholine and high-frequency EEG activity during the recovery period.

We did not find these differences in high-frequency and Ai values between the two groups during recovery period because of the fast metabolic rate of s-ketamine [2]. But we found that s-ketamine resulted in increased Ai values during sevoflurane anesthesia. Actually, the increase in BIS induced by ketamine has already been reported in patients during propofol and sevoflurane anesthesia. In the study of Vereecke HE et al*.*, BIS increase a few minutes after the administration of ketamine 0.4 mg/kg [44]. Hirota K et al*.* showed similar change of BIS after a bolus of ketamine 0.4 mg/Kg during propofol-fentanyl anesthesia [45]. During sevoflurane anesthesia, P. Hans et al*.* found the increases in BIS and entropy from 5 to 15 min after ketamine administration (0.5 mg/kg) [25]. The elevation of Ai lasting for a shorter time period in our study may be attributed to the lower dose of ketamine. The increase in BIS and Ai could be associated with the hypnotic mechanism of ketamine which mainly functions through NMDA receptors. Subanesthetic ketamine has a primary effect on NMDA receptors of inhibitory interneurons and allows downstream excitatory neurons to become disinhibited, which could induce a special EEG pattern with decreased delta and alpha waves [46–49]. Similarly, we found that subanesthetic s-ketamine induced decreased power of slow, delta, and alpha waves and increased power of beta-gamma bands during sevoflurane anesthesia. Furthermore, ketamine-induced release of cortical acetylcholine [50, 51] may also contribute to the active pattern of EEG.

However, these increases in indices of depth of anesthesia do not indicate the lightened hypnosis [25, 26, 52]. This seemingly paradoxical discrepancy between hemodynamics and EEG indices is actually the feature of ketamine or s-ketamine. This poses much challenge to anesthesia management when we rely on the EEG-derived indices during surgery. In our study, we found that heart rate and BP were not markedly changed after s-ketamine administration, although the Ai values were profoundly increased. No change of hemodynamics could be attributed to the dosage of s-ketamine we used. Previous study reported that 2 mg/kg ketamine result in a significant decrease in blood pressure during halothane anesthesia [53]. In comparison with it, we used a rather small dose of s-ketamine (0.125 mg/kg), which may not be able to substantially inhibit hemodynamics. In fact, Hans P et al*.* have found that intraoperative administration of 0.5 mg/kg ketamine do not change the hemodynamics during the sevoflurane anesthesia [25]. But of note, even at this small dose, 0.125 mg/kg s-ketamine significantly elevated the Ai value as well as the power of slow wave, delta, alpha and beta-gamma frequency of EEG, supporting the common sense that prefrontal EEG patterns are not related with the actual depth of anesthesia when using ketamine [44, 52, 54]. How the paradoxical activation of cortical neurons mediates the anesthesia of ketamine or s-ketamine is still unclear.

The administration of ketamine (≤ 1 mg/kg i.v.) during general anesthesia has beneficial effects on cognition in elderly patients undergoing major surgery [55, 56] and agitation in children and adults after rhinoplasty [9–11]. However, the effect of subanesthetic ketamine on delirium has failed to reach an agreement in clinical trials. A multicenter, international randomized trial reported that intra-operative subanesthetic ketamine (0.5 mg/kg i.v.) did not decrease delirium in older patients (> 65 years) in non-cardiac major surgery [57]. According to Hudetz JA et al., the administration of ketamine (0.5 mg/kg i.v.) during induction could attenuate postoperative delirium by 28% in cardiac surgery using cardiopulmonary bypass [58]. However, it has been reported that young and middle-aged female patients without cognitive impairment are not at a high risk for delirium [59, 60], postoperative agitation [61] or cognitive dysfunction [62–64]. Thus, no patients developed delirium, cognitive dysfunction or severe agitation in our study.

Our study has some limitations. First, only young and middle-aged female patients were recruited, which may have avoided the bias of gender and age on anesthesia emergence [31, 33, 65], but also limits the generalizability of our findings. In addition, we did not obtain blood samples or examine the plasma concentration of s-ketamine. The pharmacokinetics of s-ketamine during sevoflurane anesthesia is still not clear, and we could not obtain a dose–response relationship. It is well known that ketamine has psychotomimetic side effects, such as hallucinations, nightmares and dissociative effects [66, 67]. However, these effects were not considered in our study. Besides, the surgical stimulation and muscular activity can influence the EEG but we did not collect these data. We assumed that they were identical between the two groups. Furthermore, the sample size was relatively small, and we included only Han Chinese patients. Therefore, it is better to design a new multicenter clinical trial with a larger sample size.

## Conclusion

In conclusion, intra-operative administration of subanesthetic s-ketamine did not change the emergence time of young and middle-aged women undergoing elective laparoscopic gynecological surgery. The administration of s-ketamine can induce active EEG during sevoflurane anesthesia but did not have negative effects on neurological symptoms. Further multicenter studies with larger sample sizes are necessary to investigate whether ketamine affects the emergence time during sevoflurane anesthesia.

## Supplementary Information

**Additional file 1: Supplementary figure 1.** Blood pressure and heart rate 15min before and 20min after the administration of s-ketamine and saline. There were no significant changes in blood pressure and heart rate after the administration of subanesthetic s-ketamine. S-ketamine or saline was administered at the time point of 0.

**Additional file 2: Supplementary figure 2.** Ai values increased after the administration of s-ketamine along with the changes of EEG features. a Dynamic changes of Ai values in sevoflurane anesthesia. Subanesthetic s-ketamine or normal saline was given at 0 min. Ai values increased 5 min after the administration of s-ketamine and the increase lasted for 10 min. * indicates a difference between the s-ketamine and saline groups. b, c The correlations between the changes of Ai values and the changes in the power of slow, delta, alpha, beta-gamma waves, as well as with the alpha peak frequency.

## Data Availability

The datasets generated during the current study are not publicly available due ethical concerns but are available from the corresponding author on reasonable request.

## Abbreviations

Ai: Anesthesia index
ASA: American Society of Anesthesiologists classification
BIS: Bispectral index
BMI: Body mass index
BP: Blood pressure
BSR: Burst suppression ratio
CAM-ICU: The confusion assessment method for the Intensive Care Unit
EEG: Electroencephalogram
GABA: Gamma-aminobutyric acid
ICU: Intensive Care Unit
MAC: Minimum alveolar concentration
MMSE: Mini-mental state examination
NMDA: N-methyl-D-aspartic acid
PP: Per-protocol
SampEn: Sample entropy
SAS: Sedation-agitation scores
SD: Standard deviation
SEF: Spectral edge frequency

## References

[1] Peltoniemi MA, Hagelberg NM, Olkkola KT, Saari TI (2016). Ketamine: a review of clinical pharmacokinetics and pharmacodynamics in anesthesia and pain therapy. Clin Pharmacokinet.

[2] Zanos P, Moaddel R, Morris PJ (2018). Ketamine and ketamine metabolite pharmacology: insights into therapeutic mechanisms. Pharmacol Rev.

[3] Schmid RL, Sandler AN, Katz J (1999). Use and efficacy of low-dose ketamine in the management of acute postoperative pain: a review of current techniques and outcomes. Pain.

[4] Raeder JC, Stenseth LB (2000). Ketamine: a new look at an old drug. Curr Opin Anesthesio.

[5] Nielsen RV, Fomsgaard JS, Siegel H (2017). Intraoperative ketamine reduces immediate postoperative opioid consumption after spinal fusion surgery in chronic pain patients with opioid dependency: a randomized, blinded trial. Pain.

[6] Loftus RW, Yeager MP, Clark JA (2010). Intraoperative ketamine reduces perioperative opiate consumption in opiate-dependent patients with chronic back pain undergoing back surgery. Anesthesiology.

[7] Nielsen RV, Fomsgaard JS, Nikolajsen L, Dahl JB, Mathiesen O. Intraoperative s-ketamine for the reduction of opioid consumption and pain one year after spine surgery: a randomized clinical trial of opioid-dependent patients. Eur J Pain. 2019;23:455–60. 10.1002/ejp.1317 30246357

[8] Menigaux C, Guignard B, Fletcher D (2001). Intraoperative small-dose ketamine enhances analgesia after outpatient knee arthroscopy. Anesth Analg.

[9] Kim KM, Lee KH, Kim YH (2016). Comparison of effects of intravenous midazolam and ketamine on emergence agitation in children: randomized controlled trial. J Int Med Res.

[10] Chen JY, Jia JE, Liu TJ, Qin MJ, Li WX (2013). Comparison of the effects of dexmedetomidine, ketamine, and placebo on emergence agitation after strabismus surgery in children. Can J Anesth.

[11] Demir CY, Yuzkat N (2018). Prevention of emergence agitation with ketamine in rhinoplasty. Aesthet Plast Surg.

[12] Liu P, Li P, Li Q, et al. Effect of pretreatment of s-ketamine on postoperative depression for breast cancer patients. J Invest Surg. 2021;34:883–8. 10.1080/08941939.2019.1710626 31948296

[13] Lent JK, Arredondo A, Pugh MA, Austin PN (2019). Ketamine and treatment-resistant depression. AANA J.

[14] Mashour GA, Ben AA, Pryor KO (2018). Intraoperative ketamine for prevention of depressive symptoms after major surgery in older adults: an international, multicentre, double-blind, randomised clinical trial. Brit J Anaesth.

[15] Brinck EC, Tiippana E, Heesen M (2018). Perioperative intravenous ketamine for acute postoperative pain in adults. Cochrane Db Syst Rev.

[16] Jouguelet-Lacoste J, La Colla L, Schilling D, Chelly JE (2015). The use of intravenous infusion or single dose of low-dose ketamine for postoperative analgesia: a review of the current literature. Pain Med.

[17] Schwenk ES, Viscusi ER, Buvanendran A (2018). Consensus guidelines on the use of intravenous ketamine infusions for acute pain management from the American Society of Regional Anesthesia and Pain Medicine, the American Academy of Pain Medicine, and the American Society of Anesthesiologists. Region Anesth Pain M.

[18] Gorlin AW, Rosenfeld DM, Ramakrishna H (2016). Intravenous sub-anesthetic ketamine for perioperative analgesia. J Anaesth Clin Pharm.

[19] Atashkhoyi S, Negargar S, Hatami-Marandi P (2013). Effects of the addition of low-dose ketamine to propofol-fentanyl anaesthesia during diagnostic gynaecological laparoscopy. Eur J Obstet Gyn R B.

[20] David H, Shipp J (2011). A randomized controlled trial of ketamine/propofol versus propofol alone for emergency department procedural sedation. Ann Emerg Med.

[21] Okuyama K, Inomata S, Okubo N, Watanabe I (2011). Pretreatment with small-dose ketamine reduces predicted effect-site concentration of propofol required for loss of consciousness and laryngeal mask airway insertion in women. J Clin Anesth.

[22] Chen C, Pang Q, Tu A, Wang J, Tu F (2021). Effect of low-dose ketamine on MACBAR of sevoflurane in laparoscopic cholecystectomy: a randomized controlled trial. J Clin Pharm Ther.

[23] Hamp T, Baron-Stefaniak J, Krammel M, et al. Effect of intravenous s-ketamine on the MAC of sevoflurane: a randomised, placebo-controlled, double-blinded clinical trial. Brit J Anaesth. 2018;121:1242–8. 10.1016/j.bja.2018.08.023 30442251

[24] Carrara L, Nault M, Morisson L (2021). The impact of bolus versus continuous infusion of intravenous ketamine on bispectral index variations and desflurane administration during major surgery: the KETABIS study. Eur J Anaesth.

[25] Hans P, Dewandre PY, Brichant JF, Bonhomme V (2005). Comparative effects of ketamine on bispectral index and spectral entropy of the electroencephalogram under sevoflurane anaesthesia. Brit J Anaesth.

[26] Sengupta S, Ghosh S, Rudra A (2011). Effect of ketamine on bispectral index during propofol–fentanyl anesthesia: a randomized controlled study. Middle East J Anaesthesiol.

[27] Moro ET, Feitosa I, de Oliveira RG (2017). Ketamine does not enhance the quality of recovery following laparoscopic cholecystectomy: a randomized controlled trial. Acta Anaesth Scand.

[28] Ithnin FB, Tan D, Xu XL (2019). Low-dose S+ ketamine in target-controlled intravenous anaesthesia with remifentanil and propofol for open gynaecological surgery: a randomised controlled trial. Indian J Anaesth.

[29] Hambrecht-Wiedbusch VS, Li D, Mashour GA (2017). Paradoxical emergence: administration of subanesthetic ketamine during isoflurane anesthesia induces burst suppression but accelerates recovery. Anesthesiology.

[30] Paul R, Schaaff N, Padberg F, Moller HJ, Frodl T. Comparison of racemic ketamine and s-ketamine in treatment-resistant major depression: report of two cases. World J Biol Psychia. 2009;10:241–4. 10.1080/15622970701714370 19224412

[31] Filipescu D, Stefan M (2021). Sex and gender differences in anesthesia: relevant also for perioperative safety?. Best Prac Res-Cl Ana.

[32] Saland SK, Kabbaj M (2018). Sex differences in the pharmacokinetics of low-dose ketamine in plasma and brain of male and female rats. J Pharmacol Exp Ther.

[33] Buchanan FF, Myles PS, Cicuttini F (2011). Effect of patient sex on general anaesthesia and recovery. Brit J Anaesth.

[34] Zhang Y, Li H, Zhang X (2022). Estrogen receptor-A in medial preoptic area contributes to sex difference of mice in response to sevoflurane anesthesia. Neurosci Bull.

[35] Fu Y, Xu T, Xie K (2019). Comparative evaluation of a new depth of anesthesia index in ConView(R) system and the bispectral index during total intravenous anesthesia: a multicenter clinical trial. Biomed Res Int.

[36] Yan LM, Chen H, Yu RG (2015). Emergence agitation during recovery from intracranial surgery under general anaesthesia: a protocol and statistical analysis plan for a prospective multicentre cohort study. BMJ Open.

[37] Su X, Meng ZT, Wu XH (2016). Dexmedetomidine for prevention of delirium in elderly patients after non-cardiac surgery: a randomised, double-blind, placebo-controlled trial. Lancet.

[38] Li H, Jia J, Yang Z (2016). Mini-mental state examination in elderly Chinese: a population-based normative study. J Alzheimers Dis.

[39] Rosow CE (1997). Anesthetic drug interaction: an overview. J Clin Anesth.

[40] Hendrickx JF, Eger EN, Sonner JM, Shafer SL (2008). Is synergy the rule? A review of anesthetic interactions producing hypnosis and immobility. Anesth Analg.

[41] Abitagaoglu S, Koksal C, Alagoz S, Karip CS, Ari DE (2021). Effect of ketamine on emergence agitation following septoplasty: a randomized clinical trial. Braz J Anesthesiol.

[42] Zhang C, He J, Shi Q, Bao F, Xu J (2022). Subanaesthetic dose of esketamine during induction delays anaesthesia recovery a randomized, double-blind clinical trial. BMC Anesthesiol.

[43] Foo TY, Mohd NN, Yazid MB (2020). Ketamine-propofol (Ketofol) for procedural sedation and analgesia in children: a systematic review and meta-analysis. BMC Emerg Med.

[44] Vereecke HE, Struys MM, Mortier EP (2003). A comparison of bispectral index and ARX-derived auditory evoked potential index in measuring the clinical interaction between ketamine and propofol anaesthesia. Anaesthesia.

[45] Hirota K, Kubota T, Ishihara H, Matsuki A (1999). The effects of nitrous oxide and ketamine on the bispectral index and 95% spectral edge frequency during propofol-fentanyl anaesthesia. Eur J Anaesth.

[46] Cohen SM, Tsien RW, Goff DC, Halassa MM (2015). The impact of NMDA receptor hypofunction on GABAergic neurons in the pathophysiology of schizophrenia. Schizophr Res.

[47] Vlisides PE, Bel-Bahar T, Nelson A (2018). Subanaesthetic ketamine and altered states of consciousness in humans. Brit J Anaesth.

[48] Vlisides PE, Bel-Bahar T, Lee U (2017). Neurophysiologic correlates of ketamine sedation and anesthesia: a high-density electroencephalography study in healthy volunteers. Anesthesiology.

[49] Purdon PL, Sampson A, Pavone KJ, Brown EN (2015). Clinical electroencephalography for anesthesiologists: Part I: background and basic signatures. Anesthesiology.

[50] Pal D, Hambrecht-Wiedbusch VS, Silverstein BH, Mashour GA (2015). Electroencephalographic coherence and cortical acetylcholine during ketamine-induced unconsciousness. Brit J Anaesth.

[51] Kikuchi T, Wang Y, Shinbori H, Sato K, Okumura F (1997). Effects of ketamine and pentobarbitone on acetylcholine release from the rat frontal cortex in vivo. Brit J Anaesth.

[52] Christenson C, Martinez-Vazquez P, Breidenstein M (2021). Comparison of the Conox (qCON) and Sedline (PSI) depth of anaesthesia indices to predict the hypnotic effect during desflurane general anaesthesia with ketamine. J Clin Monit Comput.

[53] Stanley TH (1973). Blood-pressure and pulse-rate responses to ketamine during general anesthesia. Anesthesiology.

[54] Johansen JW (2006). Update on bispectral index monitoring. Best Pract Res Clin Anaesthesiol.

[55] Hudetz JA, Iqbal Z, Gandhi SD (2009). Ketamine attenuates post-operative cognitive dysfunction after cardiac surgery. Acta Anaesth Scand.

[56] Hovaguimian F, Tschopp C, Beck-Schimmer B, Puhan M (2018). Intraoperative ketamine administration to prevent delirium or postoperative cognitive dysfunction: a systematic review and meta-analysis. Acta Anaesth Scand.

[57] Avidan MS, Maybrier HR, Abdallah AB (2017). Intraoperative ketamine for prevention of postoperative delirium or pain after major surgery in older adults: an international, multicentre, double-blind, randomised clinical trial. Lancet.

[58] Hudetz JA, Patterson KM, Iqbal Z (2009). Ketamine attenuates delirium after cardiac surgery with cardiopulmonary bypass. J Cardiothor Vasc An.

[59] Mattison M (2020). Delirium. Ann Intern Med.

[60] Oh ST, Park JY (2019). Postoperative delirium. Korean J Anesthesiol.

[61] Lee SJ, Sung TY (2020). Emergence agitation: current knowledge and unresolved questions. Korean J Anesthesiol.

[62] Kotekar N, Shenkar A, Nagaraj R (2018). Postoperative cognitive dysfunction - current preventive strategies. Clin Interv Aging.

[63] Rundshagen I (2014). Postoperative cognitive dysfunction. Dtsch Arztebl Int.

[64] Evered LA, Silbert BS (2018). Postoperative cognitive dysfunction and noncardiac surgery. Anesth Analg.

[65] Misal US, Joshi SA, Shaikh MM (2016). Delayed recovery from anesthesia: a postgraduate educational review. Anesth Essays Res.

[66] Han E, Kwon NJ, Feng LY, Li JH, Chung H (2016). Illegal use patterns, side effects, and analytical methods of ketamine. Forensic Sci Int.

[67] Niesters M, Martini C, Dahan A (2014). Ketamine for chronic pain: risks and benefits. Brit J Clin Pharmaco.

