# Supplementary material for: Effects of intra-operative administration of subanesthetic s-ketamine on emergence from sevoflurane anesthesia: a randomized double-blind placebo-controlled study
Source: BMC Anesthesiol. 2023 Jun 23;23:221. doi: 10.1186/s12871-023-02170-5 (PMC10288804; doi:10.1186/s12871-023-02170-5)
Supplement: Supplementary file 1 — Additional file 1: Supplementary figure 1. Blood pressure and heart rate 15min before and 20min after the administration of s-ketamine and saline. There were no significant changes in blood pressure and heart rate after the administration of subanesthetic s-ketamine. S-ketamine or saline was administered at the time point of 0. [file 12871_2023_2170_MOESM1_ESM.docx]

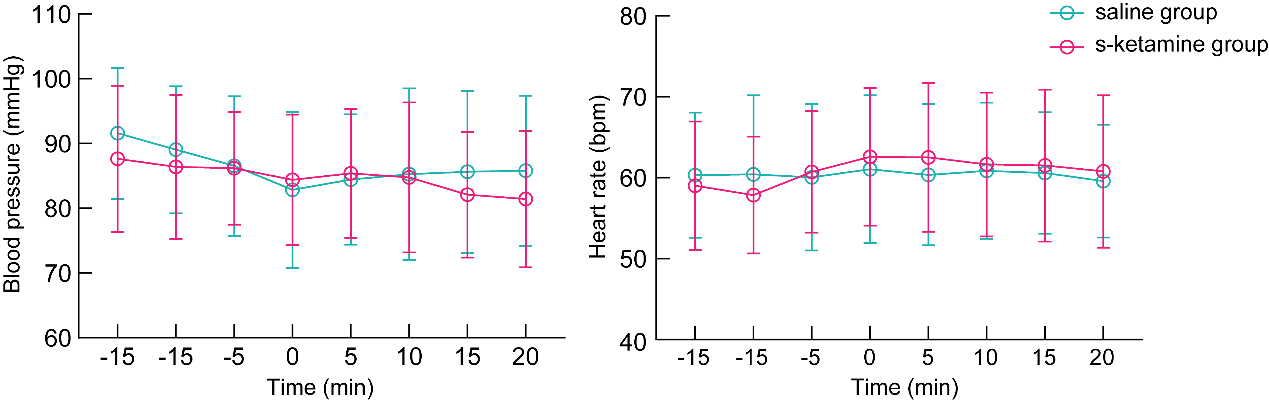


**Supplementary figure 1.** **Blood pressure and heart rate 15min before and 20min after the administration of s-ketamine and saline.** There were no significant changes in blood pressure and heart rate after the administration of subanesthetic s-ketamine. S-ketamine or saline was administered at the time point of 0.
