# Supplementary material for: Effects of intra-operative administration of subanesthetic s-ketamine on emergence from sevoflurane anesthesia: a randomized double-blind placebo-controlled study
Source: BMC Anesthesiol. 2023 Jun 23;23:221. doi: 10.1186/s12871-023-02170-5 (PMC10288804; doi:10.1186/s12871-023-02170-5)
Supplement: Supplementary file 2 — Additional file 2: Supplementary figure 2. Ai values increased after the administration of s-ketamine along with the changes of EEG features. a Dynamic changes of Ai values in sevoflurane anesthesia. Subanesthetic s-ketamine or normal saline was given at 0 min. Ai values increased 5 min after the administration of s-ketamine and the increase lasted for 10 min. * indicates a difference between the s-ketamine and saline groups. b, c The correlations between the changes of Ai values and the changes in the power of slow, delta, alpha, beta-gamma waves, as well as with the alpha peak frequency. [file 12871_2023_2170_MOESM2_ESM.docx]

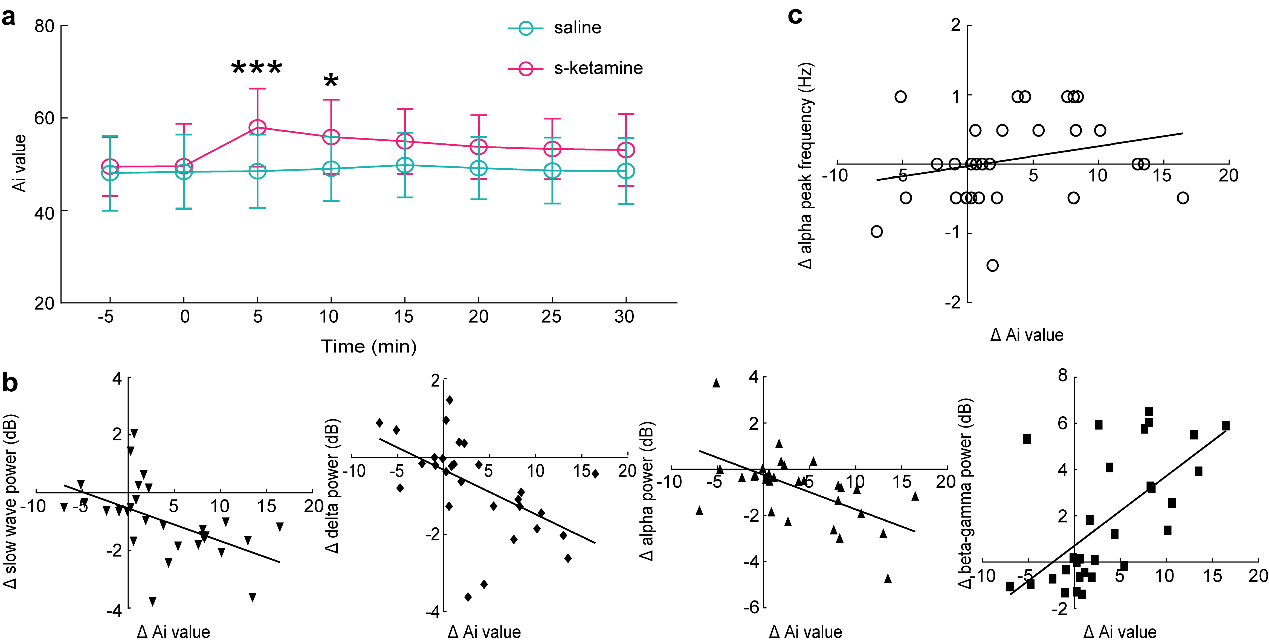


**Supplementary figure 2.** **Ai values increased after the administration of s-ketamine along with the changes of EEG features.** **a** Dynamic changes of Ai values in sevoflurane anesthesia. Subanesthetic s-ketamine or normal saline was given at 0 min. Ai values increased 5 min after the administration of s-ketamine and the increase lasted for 10 min. * indicates a difference between the s-ketamine and saline groups. **b, c** The correlations between the changes of Ai values and the changes in the power of slow, delta, alpha, beta-gamma waves, as well as with the alpha peak frequency.
